# Risk factors for type 2 diabetes mellitus among university students in Saudi Arabia: a multi-regional cross-sectional study

**DOI:** 10.3389/fendo.2026.1904706

**Published:** 2026-07-28

**Authors:** Abdulrahman I. Alaqil, Fahad Bin Radhyan

**Affiliations:** 1 College of Education, Department of Physical Education, King Faisal University, Al-Ahsa, Saudi Arabia; 2 College of General Studies, Department of Physical Education, King Fahd University of Petroleum and Minerals, Dhahran, Saudi Arabia

**Keywords:** AUSDRISK, obesity, physical activity, risk factors, Saudi Arabia, type 2 diabetes mellitus, university students, youth

## Abstract

**Background:**

Currently, 28% of Saudi Arabian adults have type 2 diabetes mellitus (T2DM), projected to exceed 50% by 2030. University students remain largely unscreened despite evidence of substantial T2DM risk during this critical lifestyle transition period.

**Aim:**

This multi-regional study investigated the relationship between modifiable and non-modifiable risk factors and elevated AUSDRISK-defined T2DM risk among Saudi university students.

**Methods:**

A sample of 564 students from three regions of Saudi Arabia was selected using systematic random sampling across academic disciplines and levels. T2DM risk was assessed using a modified eight-item Australian Type 2 Diabetes Risk Assessment Tool (AUSDRISK; low ≤5, intermediate 6–11, high ≥12). Variables examined included sociodemographic characteristics, BMI (WHO classification), tobacco use, physical activity (IPAQ-adapted), sedentary behavior, and dietary intake (food frequency questionnaire). Binary logistic regression provided adjusted odds ratios (aOR) with 95% confidence intervals (CI) for factors independently associated with elevated AUSDRISK classification (score ≥6).

**Results:**

Around a third (32.4%) of participants (N = 564; 75.9% female; mean BMI 22.2 ± 4.0 kg/m²) had elevated AUSDRISK-defined T2DM risk (mean score 4.68 ± 2.70). Males had significantly higher risk than females (75.7% vs. 18.7%; p < 0.001). All overweight and obese participants (100%) were at elevated risk versus 16.0% of normal-weight peers. Bivariate analysis (n = 564) identified sex, BMI, smoking, physical activity, academic level, and dietary intake as significantly associated with elevated AUSDRISK classification (all p < 0.05). In the logistic regression restricted to non-AUSDRISK predictors (n = 558), income (aOR = 0.88, 95% CI 0.78–0.99, p = 0.033), smoking (aOR = 2.62, 95% CI 1.38–4.97, p = 0.003), sedentary behavior (aOR = 0.79, 95% CI 0.62–0.99, p = 0.041; interpreted cautiously due to potential residual confounding by sex), and academic level (aOR = 0.83, 95% CI 0.69–0.99, p = 0.039) were all independently significant. Sex, BMI, and physical activity are structural AUSDRISK components presented descriptively only.

**Conclusion:**

Nearly one in three Saudi university students had elevated T2DM risk. The findings underline the urgent need for university-based, sex-specific, socioeconomically targeted interventions incorporating BMI screening, physical activity promotion, and dietary education.

## 1 Introduction

Type 2 diabetes mellitus (T2DM) represents one of the most significant non-communicable disease epidemics worldwide. In 2021, it affected approximately 537 million adults globally; this is projected to rise to 700 million by 2045 (1–3). Over 90% of all diabetes cases are classified as T2DM, with these cases driven by a combination of non-modifiable genetic factors and modifiable lifestyle behaviors (4–7). The Eastern Mediterranean region bears a disproportionate T2DM burden, having the second-highest prevalence of diabetes worldwide (8). Saudi Arabia is among the most severely affected countries, with a pooled, adult T2DM prevalence of 28%, based on a recent systematic review and meta-analysis (2, 4, 9), ranking Saudi Arabia as having the second highest T2DM prevalence in the Middle East and seventh globally. Between 1990 and 2019, Global Burden of Disease data illustrate that the age-standardized incidence and prevalence of T2DM in Saudi Arabia increased from 63.5% to 75.7%, respectively, with a notable rise in the proportion of burden borne by those under 40 years of age (10). If current trends continue, over 50% of Saudi adults are projected to develop diabetes by 2030 (4), with an unusually high prevalence of T2DM among Arab children and under-18s (1, 11). Besides the clinical burden of T2DM, the disease imposes severe financial strain on Arab healthcare systems: The cost of care for T2DM patients is among the highest globally, and affordability is a critical barrier to effective management (1, 12). This alarming trend has been linked to rapid urbanization, widespread physical inactivity, the adoption of westernized dietary habits, increased tobacco use, and persistently poor public awareness about T2DM risk factors (1, 13–18).

T2DM has been historically linked with middle-aged and older populations; however, its early onset is increasingly prevalent among younger populations, including university students (19–21). Attending university represents a critical transitional period for young people, during which they may adopt new, unhealthy lifestyle behaviors that elevate T2DM risk, such as physical inactivity, poor dietary choices, prolonged sedentary behavior, smoking, and disrupted sleep (4, 16, 17, 22, 23). International studies have consistently identified sedentariness as the most prevalent modifiable risk factor for T2DM among university students, followed by overweight, central obesity, and elevated fasting glucose (19, 21, 23). A recent multinational study of 2,787 university students in Egypt, Saudi Arabia, and Yemen found that T2DM risk was independently associated with male sex, older age, and higher BMI, with marked differences in risk profiles across countries (24). In the Saudi context specifically, one study reported that over 60% of university students at Hail University were at intermediate to high risk of T2DM, with male students at significantly greater risk than females (4). A national-level Saudi study identified three independent sociodemographic predictors of T2DM risk: marital status, city size, and employment status (2). Another study identified three significant anthropometric risk markers among Saudi university students: elevated BMI, increased waist circumference, and high visceral fat (13). Dietary behaviors and physical activity levels play a central role in T2DM risk for Saudi Arabians, with low physical activity, high consumption of soft drinks and fast food, and inadequate fruit and vegetable intake significantly associated with overweight, obesity, and metabolic syndrome risk (14, 17, 25–27). Despite positive attitudes toward the prevention of T2DM, knowledge of its risk factors among Saudi college students remains poor. Less than 15% of students demonstrated good T2DM risk factor knowledge scores in one study (18), and many students underestimate their personal risk of T2DM despite having multiple established risk factors (20, 28).

Despite the evidence that T2DM risks are rising among the Saudi university student population, there is a lack of comprehensive multi-university studies across different regions of Saudi Arabia that simultaneously examine the full spectrum of modifiable and non-modifiable T2DM risk factors, including detailed dietary intake, physical activity, sedentary behavior, tobacco use, and anthropometric and sociodemographic characteristics (2, 4, 24). The findings of most existing studies on this topic have limited generalizability as they investigated T2DM risk factors in single institutions, single risk domains, or specific subgroups (13, 17, 18). Furthermore, in light of the projected doubling of diabetes cases across the Arab nations by 2035 (1), the growth of the T2DM burden among the under-40s in Saudi Arabia (10), and the substantial economic costs of dealing with this epidemic already borne by regional healthcare systems (1), identifying high-risk T2DM behavioral profiles among Saudi university students is a critical and time-sensitive public health priority. Gaining a good understanding of the combined association between these T2DM risk factors is essential to enable the creation of targeted, evidence-based prevention programs for this vulnerable population (14, 20, 21, 23). To address this gap in the literature, the current study aimed to investigate the association between modifiable and non-modifiable T2DM risk factors among university students across Saudi Arabia to provide comprehensive baseline data to support national diabetes prevention efforts.

## 2 Methods

### 2.1 Study design, setting

This cross-sectional study was conducted between March and May 2023. A self-administered, online Google Forms questionnaire was sent to the target population via their university email by the Deanship of Scientific Research. Participation was voluntary and anonymous. Informed consent was obtained electronically: participants were presented with a participant information sheet at the start of the questionnaire, and submission of the completed questionnaire constituted electronic informed consent, as approved by the Institutional Review Board. Ethical approval was obtained from the Institutional Review Board of King Faisal University, Saudi Arabia (KFU-REC-2022-OCT-ETHICS202). The study was conducted in accordance with the Declaration of Helsinki.

### 2.2 Study participants and sampling

The target population comprised students enrolled at Saudi Arabian universities aged > 18. The sample included university students across three regions of Saudi Arabia: Makkah (n = 220; 39.0%), Eastern Province (n = 166; 29.4%), Riyadh (n = 161; 28.5%), as well as other regions (n=17; 3.0%). Three universities participated (one per region: Makkah, Eastern Province, and Riyadh). Three colleges per university were selected using systematic random sampling, yielding nine colleges in total. Of these, participants from three academic domains (health and medical sciences, arts and humanities, and science and engineering) were selected. Within each college, one male and one female class from six academic levels (undergraduates: four levels; postgraduates: two levels) were invited to participate. Students who indicated a self-reported diagnosis of T2DM on the comorbidities checklist were excluded from all analyses to avoid confounding. Six participants (1.1%) were excluded on this basis, yielding a final analytic sample of n = 558. A minimum sample size of 384 was estimated based on a 50% expected prevalence of T2DM risk, 5% margin of error, and 95% confidence level; the final sample of 564 exceeded this requirement. Reporting followed the Strengthening the Reporting of Observational Studies in Epidemiology (STROBE) guidelines for cross-sectional studies; a completed STROBE checklist is provided as **Supplementary Material 1**. All students enrolled in the selected classes (n = 564) were invited to participate via their institutional email. A participation rate of 100% was recorded among the selected classes; all 564 enrolled students submitted the questionnaire. This high rate was attributed to class-level coordination by the Deanship of Scientific Research. The authors acknowledged that the number of students who received, opened, or accessed the invitation link cannot be independently verified, and this should be considered when interpreting the participation rate. Questionnaire submission confirmed participation. The final sample who completed the questionnaire comprised a total of 564 students (males: n = 136; females: n = 428).

### 2.3 Data collection instrument

A structured, Arabic-language version of the eight-item Australian Type 2 Diabetes Risk Assessment Tool (AUSDRISK) questionnaire was created to appraise the following factors: (i) sociodemographic and anthropometric; (ii) tobacco use; (iii) physical activity and sedentary behavior; and (iv) dietary habits, using an adapted semi-quantitative food frequency questionnaire (SFFQ) validated for use with Saudi populations (29). Of the 110 food items, daily vegetable and daily fruit intake were selected *a priori* for bivariate analysis as these were the two dietary items explicitly scored in the modified AUSDRISK instrument; full FFQ analysis against objective metabolic outcomes was planned for future work. The questionnaire included 110 food items with four frequency response categories (never or less than once per month, 1–3 times/week, 4–6 times/week, and daily), as well as an open-ended item for reporting unlisted foods (29).

### 2.4 Measures

#### 2.4.1 Outcome variable: T2DM Risk Score (Modified AUSDRISK)

The outcome (T2DM risk score) was assessed using an Arabic-language version of AUSDRISK, a validated self-report screening instrument previously used with Saudi and Arab student populations (4, 24). As a dedicated family history of diabetes was not included in the questionnaire, a modified eight-item AUSDRISK was used (**Table 1**). As waist circumference was not measured, BMI category was substituted for it, a method used in comparable prior studies (4). The results comprised total T2DM risk scores ranging from 0 to 23 (theoretical maximum), categorized as low risk (≤5), intermediate risk (6-11), or high risk (≥12).

**Table 1:** Modified AUSDRISK scoring criteria applied in this study.

AUSDRISK item	Criterion/response	Points
Age	Under 35 years	0
Sex	Male/Female	3/0
Ethnicity	Arab/Asian	2
History of high blood glucose	Yes/No	6/0
Antihypertensive medication	Yes/No	2/0
Physical activity	< 150/≥150 min/week	2/0
Daily vegetable/fruit intake	Not every day/Every day	1/0
BMI (waist substitute)	< 25.0/25.0–29.9/≥30 kg/m²	0/4/7
Family history of diabetes	*Not assessed — study limitation*	*—*
Risk categories	Low: ≤5 | Intermediate: 6–11 | High: ≥12	Max: 23

BMI, body mass index; AUSDRISK, Australian Type 2 Diabetes Risk Assessment Tool. The family history item (maximum 3 points in the original tool) was omitted due to questionnaire design and is noted as a study limitation.

#### 2.4.2 Physical activity and sedentary behavior

Physical activity was assessed using items adapted from the short form version of the International Physical Activity Questionnaire (IPAQ-SF). This instrument collected data on participants’ reported frequency of engagement in physical activity (days per week) and duration (minutes per session) for vigorous-intensity activity, moderate-intensity activity, and walking over the preceding seven days. Total weekly physical activity volume (minutes/week) was estimated by converting ordinal response categories to their midpoint values and summing them to compute the AUSDRISK scores. As per WHO physical activity guidelines, participants achieving ≥150 minutes/week of physical activity were classified as sufficiently active (0 points), while those reporting physical activity below this threshold were deemed insufficiently active (2 points). Sedentary behavior was assessed by summing self-reported daily sitting time across six situational contexts (work/study, homework, television, mobile phone use, laptop use, and gaming), with ordinal responses converted to midpoint hours (<8 h/day), moderately sedentary (8–12 h/day), or highly sedentary (>12 h/day) (30). Participants’ sedentariness was then classified as low (<8 h/day), moderate (8–12 h/day), or high (>12 h/day).

### 2.5 Statistical analysis

Data were analyzed using IBM’s SPSS Statistics package (version 29.0, IBM Corp., Armonk, NY, USA). Continuous variables were reported as mean ± SD or medians (IQR); categorical variables were reported as frequencies and percentages (%). For regression analyses, participants with intermediate and high T2DM risk scores were combined into an ‘elevated risk’ group (score ≥ 6), while those scoring ≤ 5 were assigned to the ‘low risk’ group. Bivariate associations between each predictor and elevated T2DM risk were examined using Pearson chi-square tests; Fisher’s exact test was applied when expected cell counts were < 5. Independent samples t-tests and one-way ANOVA were used to compare mean across-group AUSDRISK scores. Variables with p < 0.25 at the bivariate level were eligible for multivariable modeling. To avoid circular inference, since sex, BMI, physical activity, and dietary intake are direct components of the AUSDRISK score, the logistic regression model was restricted to variables NOT embedded in the AUSDRISK score: monthly family income, smoking status, sedentary behavior category, and academic level. All four variables were retained in the final adjusted model. Crude and adjusted odds ratios (OR/aOR) with 95% confidence intervals (CI) are reported. Multicollinearity was assessed using variance inflation factors (VIF); model fit was evaluated using the Hosmer–Lemeshow test, Nagelkerke R², and overall classification accuracy. A two-tailed p < 0.05 was considered statistically significant.

## 3 Results

The total recruited sample comprised of 564 participants. Six participants who self-reported a T2DM diagnosis were excluded from all analyses, yielding a final analytic sample of n = 558. Descriptive characteristics were reported for the full recruited sample (n = 564) unless otherwise stated. Of the 564 participants, 75.9% were female and 24.1% male, with a mean age of 21.2 ± 3.1 years. Most were Saudi nationals (97.9%), single (93.4%), and from three regions: Makkah (39.0%), Eastern Province (29.4%), and Riyadh (28.5%). Participants self-reported their body weight (kg) and height (cm), from which BMI was calculated. Mean BMI was 22.2 ± 4.0 kg/m²; 18.1% were overweight or obese. Current smoking status was 7.6%. Physical activity was high (≥300 min/week) in 68.4% of participants, while 17.0% were insufficiently active (<150 min/week). Over half (57.4%) of participants were highly sedentary (>12 h/day) with a mean of 14.2 ± 7.5 h/day. Comorbidities were rare in this young sample: six participants (1.1%) reported T2DM on the comorbidities checklist and were subsequently excluded from all analyses (final n = 558); hypertension 0.2% (see **Table 2**).

**Table 2:** Characteristics of the sample population (n=564).

Characteristic	n	%
Sex		
Male	136	24.1
Female	428	75.9
Age group (years)		
< 20	164	29.1
20–25	385	68.3
> 25	15	2.7
Region		
Makkah	220	39.0
Eastern Province	166	29.4
Riyadh	161	28.5
Other	17	3.0
Monthly family income (SAR)		
≤ 3,000	101	17.9
3,001–8,000	128	22.7
8,001–13,000	129	22.9
13,001–18,000	85	15.1
18,001–23,000	62	11.0
≥ 23,000	59	10.5
**BMI category**	Mean ± SD: 22.2 ± 4.0 kg/m²	
Underweight (< 18.5)	93	16.5
Normal weight (18.5–24.9)	363	64.4
Overweight (25.0–29.9)	75	13.3
Obese (≥ 30.0)	27	4.8
**Current smoker**	43	7.6
**Physical activity min/week**	Median (IQR): 495 (240–855) min/week	
Low (< 150 min/week)	96	17.0
Moderate (150–299 min/week)	82	14.5
High (≥ 300 min/week)	386	68.4
**Sedentary behavior h/day**	Mean ± SD: 14.2 ± 7.5 h/day	
Low (< 8 h/day)	100	17.7
Moderate (8–12 h/day)	140	24.8
High (> 12 h/day)	324	57.4

BMI = body mass index; IQR = interquartile range; SAR = Saudi Riyals.

The mean AUSDRISK score was 4.68 ± 2.70, with an observed range of 2–17 and a theoretical maximum of 23 points after omission of the family history item. Overall, 67.6% were at low risk (≤5), 29.3% at intermediate risk (6-11), and 3.2% at high risk (≥12), resulting in an elevated T2DM risk prevalence of 32.4% (n = 183). Males scored markedly higher than females (6.99 ± 2.46 vs 3.94 ± 2.34; t = 13.04, p<0.001), with 75.7% of males versus 18.7% of females at elevated risk of T2DM. All overweight and obese participants (100%) were classified as elevated risk, compared to 16.0% of normal-weight students (See **Table 3**).

**Table 3:** AUSDRISK Risk Category by Key Variables.

Variable	Low n (%)	Intermediate n (%)	High n (%)	p-value
Overall (n=564)	381 (67.6)	165 (29.3)	18 (3.2)	
Sex				< 0.001
Male (n=136)	33 (24.3)	93 (68.4)	10 (7.4)	
Female (n=428)	348 (81.3)	72 (16.8)	8 (1.9)	
BMI category				< 0.001
Underweight (n=93)	72 (77.4)	20 (21.5)	1 (1.1)	
Normal weight (n=363)	305 (84.0)	55 (15.2)	3 (0.8)	
Overweight (n=75)	0 (0.0)	73 (97.3)	2 (2.7)	
Obese (n=27)	0 (0.0)	5 (18.5)	22 (81.5)	
Smoking status				0.011
Non-smoker (n=521)	360 (69.1)	147 (28.2)	14 (2.7)	
Current smoker (n=43)	21 (48.8)	18 (41.9)	4 (9.3)	
Physical activity				0.006
Low (n=96)	52 (54.2)	40 (41.7)	4 (4.2)	
Moderate (n=82)	54 (65.9)	26 (31.7)	2 (2.4)	
High (n=386)	275 (71.2)	99 (25.6)	12 (3.1)	
Academic level				0.001
UG Year 1 (n=97)	50 (51.5)	44 (45.4)	3 (3.1)	
UG Year 2 (n=188)	138 (73.4)	45 (23.9)	5 (2.7)	
UG Year 3 (n=172)	115 (66.9)	52 (30.2)	5 (2.9)	
UG Year 4 (n=96)	72 (75.0)	21 (21.9)	3 (3.1)	

p-values from chi-square tests. Low ≤5, Intermediate 6–11, High ≥12. UG = undergraduate.

Bivariate analysis (see **Table 4**) identified sex (χ² = 150.62, p < 0.001), BMI category (χ² = 261.45, p < 0.001), smoking (χ² = 6.54, p = 0.011), physical activity (χ² = 10.36, p = 0.006), academic level (χ² = 19.90, p = 0.001), daily vegetable intake (χ² = 19.56, p < 0.001), and daily fruit intake (χ² = 6.79, p = 0.009) as significantly associated with elevated T2DM risk. Monthly income showed a borderline inverse association (χ² = 11.03, p = 0.051). Nationality and marital status were not significant (both p > 0.05).

**Table 4:** Association Between Risk Factors and Elevated T2DM Risk among study participants.

Variable	Low risk n=381 (%)	Elevated risk n=183 (%)	χ²	p-value
Sex			150.62	< 0.001
Male (n=136)	33 (24.3)	103 (75.7)		
Female (n=428)	348 (81.3)	80 (18.7)		
BMI category			261.45	< 0.001
Underweight (n=93)	72 (77.4)	21 (22.6)		
Normal weight (n=363)	305 (84.0)	58 (16.0)		
Overweight (n=75)	0 (0.0)	75 (100.0)		
Obese (n=27)	0 (0.0)	27 (100.0)		
Smoking status			6.54	0.011
Non-smoker (n=521)	360 (69.1)	161 (30.9)		
Current smoker (n=43)	21 (48.8)	22 (51.2)		
Physical activity			10.36	0.006
Low < 150 min/week (n=96)	52 (54.2)	44 (45.8)		
Moderate 150–299 (n=82)	54 (65.9)	28 (34.1)		
High ≥ 300 min/week (n=386)	275 (71.2)	111 (28.8)		
Monthly income (SAR)			11.03	0.051
≤ 3,000 (n=101)	56 (55.4)	45 (44.6)		
3,001–8,000 (n=128)	84 (65.6)	44 (34.4)		
≥ 8,001 (n=335)	241 (71.9)	94 (28.1)		
Daily vegetable intake			19.56	< 0.001
Yes (n=152)	125 (82.2)	27 (17.8)		
No (n=412)	256 (62.1)	156 (37.9)		
Daily fruit intake			6.79	0.009
Yes (n=75)	61 (81.3)	14 (18.7)		
No (n=489)	320 (65.4)	169 (34.6)		

Elevated risk, intermediate (6–11) or high (≥12) AUSDRISK. χ², chi-square statistic. SAR, Saudi Riyals.

To address the circularity between AUSDRISK component variables and the AUSDRISK-derived outcome, the logistic regression was restricted to variables not embedded in the AUSDRISK score (see **Table 5**). Six participants were excluded due to missing income data (final n = 558). Among non-AUSDRISK predictors (n = 558), four variables were entered into the adjusted logistic regression model: monthly family income, smoking status, sedentary behavior, and academic level. All four were independently significant after adjustment. Higher monthly family income was associated with lower odds of elevated AUSDRISK classification (aOR = 0.88, 95% CI 0.78–0.99, p = 0.033). Current smoking was associated with higher odds (aOR = 2.62, 95% CI 1.38–4.97, p = 0.003). A higher sedentary behavior category was inversely associated with elevated risk (aOR = 0.79, 95% CI 0.62–0.99, p = 0.041). Higher academic level was associated with lower odds of elevated AUSDRISK classification (aOR = 0.83, 95% CI 0.69–0.99, p = 0.039). Model fit: Nagelkerke R² = 0.055; accuracy = 68.6%; sensitivity = 7.9%; specificity = 96.9%; all VIF < 1.02. The distributions of AUSDRISK risk classification by AUSDRISK-component variables (sex, BMI, physical activity, diet) are presented descriptively in **Table 3** and **4**.

**Table 5:** Non-AUSDRISK Predictors of Elevated AUSDRISK Classification (n = 558).

Variable	Crude OR (95% CI)	p	Adjusted OR (95% CI)	p	VIF
Monthly income (per unit ↑)	0.86 (0.77–0.97)	0.014	0.88 (0.78–0.99)	0.033	1.01
Current smoker (ref: non-smoker)	2.43 (1.30–4.55)	0.005	2.62 (1.38–4.97)	0.003	1.01
Sedentary behavior (per unit ↑)	0.78 (0.62–0.97)	0.028	0.79 (0.62–0.99)	0.041	1.01
Academic level (per unit ↑)	0.83 (0.69–0.99)	0.034	0.83 (0.69–0.99)	0.039	1.01

OR = odds ratio; CI = confidence interval; VIF = variance inflation factor. Income: 1 (lowest)–6 (highest). Sedentary behavior: 0=low (<8 h/day), 1=moderate (8–12 h/day), 2=high (>12 h/day). Academic level: 1=UG Year 1 through 6=PhD. Nagelkerke R² = 0.055; accuracy = 68.6%; sensitivity = 7.9%; specificity = 96.9%. All VIF < 1.02.

## 4 Discussion

Using data collected with a modified version of the AUSDRISK instrument, this multi-institutional, cross-sectional study found that the Saudi university students sampled had an elevated prevalence of T2DM risk of 32.4%. For comparison, this ure is notably lower than the 61.9% T2DM risk prevalence reported among Hail University students using the same instrument (4), and the 46.2% documented in a recent Saudi multi-institution study (31). These differences may be attributable in part to the predominantly female composition of the sample in the current study (75.9%), given that female sex contributes zero points to the AUSDRISK score. T2DM risk prevalence scores vary widely across studies of Arab student populations (from 14–62%); these differences reflect heterogeneity in sample composition, research instrument choice, and institutional context (2, 4, 24). Despite these methodological variations, the current findings affirm that Saudi university-age adults, a population historically under-screened despite bearing a growing share of the national diabetes burden, have a substantial T2DM risk (1, 9). The current study makes a significant contribution to the evidence base on early-onset T2DM by providing multi-regional, systematically sampled data from three of Saudi Arabia’s most populated regions.

Male sex showed the strongest descriptive association with elevated AUSDRISK classification: 75.7% of males versus 18.7% of females were classified as elevated risk (p < 0.001), reflecting both the structural contribution of the sex item to the AUSDRISK scoring (3 points for males vs. 0 for females) and the higher prevalence of overweight and obesity among male students in the current sample. As sex was a direct component of the AUSDRISK score, no adjusted odds ratio was reported for sex in the revised regression model; the observed group difference was therefore interpreted descriptively only. Biologically, males preferentially accumulate visceral adipose tissue, which was mechanistically linked to hepatic insulin resistance, dyslipidemia, and impaired beta-cell function at lower absolute adiposity levels than females (32, 33). Visceral fat area has been identified as a sex-specific factor associated with elevated AUSDRISK classification of T2DM incidence, with markedly different optimal cut-offs between males and females (34). In the Saudi sociocultural context, male students are more likely to consume fast food, sugary beverages, and calorie-dense diets while exhibiting less concern for weight management than female counterparts (13, 17, 18, 35). Consistent with the current findings, male students comprised 85.7% of those at intermediate or high risk of T2DM in a previous study (4), and were identified as the highest-risk group across three Arab countries (24). This reinforces sex as the dominant demographic predictor of T2DM risk in this population, underscoring the urgent need for prevention initiatives targeting males specifically. As sex, BMI, physical activity, and daily fruit/vegetable intake are direct components of the AUSDRISK score, group differences across these variables reflect, in part, the scoring structure of the instrument itself and should not be interpreted as independent etiological predictors. The findings were more appropriately described as characterizing the distribution of AUSDRISK-defined risk across key population subgroups.

BMI category showed the strongest descriptive association with elevated AUSDRISK classification: 100% of overweight and obese participants were classified as elevated risk compared with 16.0% of their normal-weight peers, reflecting a clear dose-response gradient. As BMI is a direct component of the AUSDRISK score (contributing 0, 4, or 7 points), this association was structural rather than independent; overweight/obese participants cannot score below the elevated risk threshold. Nevertheless, the biological basis for this association was well-established: This finding was biologically grounded in the established pathway whereby excess adiposity promotes insulin resistance through ectopic lipid deposition, adipokine dysregulation, and chronic low-grade inflammation (36). Critically, emerging evidence suggests that the standard WHO BMI obesity threshold (≥30 kg/m²) underestimates T2DM risk in Arab populations: a large population-based cohort study in England identified a BMI cut-off of 26.6 kg/m² as the threshold at which Arabs face equivalent T2DM incidence risk compared to non-Arab (white) populations at 30.0 kg/m², substantially lower than the conventional WHO criterion (37). This ethnicity-specific threshold implies that the proportion of Saudi university students at meaningful metabolic risk due to adiposity was likely greater than suggested by the standard BMI categorization, and that even normal-weight students in this study may harbor excess cardiometabolic risk (38). The combined prevalence of overweight and obesity in our sample (18.1%) was broadly consistent with other Saudi studies on university students (18–36%) (13, 17, 39). Modeling data suggests that a 5% reduction in obesity in the Saudi population could avert over 550,000 cases of T2DM by 2030 (39), underscoring the transformative potential of population-level weight management interventions.

Higher physical activity was descriptively associated with lower rates of elevated AUSDRISK classification: 45.8% of low-active students were at elevated risk versus 28.8% of highly active students (p = 0.006). As physical activity is a direct AUSDRISK scoring component, this gradient reflects the instrument’s design. Nonetheless, the protective role of physical activity against T2DM is well-established independently: A systematic review and dose-response meta-analysis of prospective cohort studies found a 26% risk reduction for T2DM among those achieving 150 min/week of moderate-intensity activity relative to inactive individuals (40). Importantly, 17.0% of the current sample reported low levels of physical activity (<150 min/week), which is substantially lower than the 61.4% reported by Antwi et al. (2020) in a US college sample (23) and the 34.7% in a Saudi college sample (4). However, the notably high activity levels in the current sample may be due to self-report overestimation, a recognized limitation of the IPAQ short form, and therefore should be interpreted with caution (41). Even so, these findings reinforce the importance of promoting regular physical activity through structured university-based programs, as recommended by WHO physical activity guidelines (42).

In the non-AUSDRISK logistic regression model (restricted to predictors external to the AUSDRISK score), four variables were independently significant after adjustment: income, smoking, sedentary behavior, and academic level. Higher monthly family income was associated with lower odds of an elevated AUSDRISK classification (aOR = 0.88, 95% CI 0.78–0.99, p = 0.033), as income was not incorporated into the AUSDRISK scoring algorithm. This finding was consistent with robust global epidemiological evidence: one systematic review found that low income was associated with a 40% higher relative risk of T2DM (RR = 1.40, 95% CI 1.04–1.88) compared with high income (43). This aligned with a large Taiwanese cohort study that identified a significantly higher risk of early-onset T2DM among youth from lower-income families (43). In the Saudi context, the high elevated AUSDRISK-defined T2DM risk among lower-income students may be due to restricted access to healthier food options, recreational facilities, and healthcare resources, which increase their exposure to obesogenic environments (2, 44). Taken together, these findings suggest that T2DM prevention programs should be socioeconomically targeted to pay particular attention to students from lower-income backgrounds.

Bivariate analysis identified significant associations between inadequate daily intake of vegetables and fruit and elevated T2DM risk (p < 0.001 and p = 0.009, respectively): 73.0% of participants do not consume vegetables daily, and 86.7% do not consume fruit daily. These rates markedly exceed those observed in Western student populations and are consistent with documented patterns among Saudi university students, where convenience, palatability, and time constraints drive the frequent consumption of ultra-processed and fast foods at the expense of plant-based foods (4, 45). Inadequate fruit and vegetable intake was mechanistically linked to increased oxidative stress, inflammation, and micronutrient deficiencies that impair insulin sensitivity and accelerate T2DM pathogenesis (1). Although dietary variables were not entered into the regression model to avoid overfitting, the dietary associations observed reinforce the importance of campus-based nutrition education and structural improvements to food environments, including providing better access to affordable healthy food options within university settings.

In the non-AUSDRISK logistic regression model, current smoking was independently associated with higher odds of elevated AUSDRISK classification (aOR = 2.62, 95% CI 1.38–4.97, p = 0.003). A higher sedentary behavior category was inversely associated with elevated AUSDRISK classification (aOR = 0.79, 95% CI 0.62–0.99, p = 0.041), though this association should be interpreted cautiously as the majority of highly sedentary students were female, who attract lower AUSDRISK scores due to the sex scoring component. Higher academic level was also independently associated with lower odds of elevated AUSDRISK classification (aOR = 0.83, 95% CI 0.69–0.99, p = 0.039). These findings align with Alsulami et al. (2023), who confirmed that smoking was associated with cardiometabolic risk in Saudi university students (17). These findings underscore the importance of tobacco cessation and sedentary behavior reduction as T2DM prevention priorities, supported by robust evidence of their cardiometabolic effects beyond the pathways captured by the AUSDRISK scoring system (1).

This study has several strengths, including its multi-university, multi-region design covering three major regions of Saudi Arabia, a systematic random sampling strategy to ensure representation across academic disciplines and levels, and a validated T2DM risk assessment instrument. However, eight limitations should be acknowledged. First, its cross-sectional design precludes causal inference regarding the association between modifiable and non-modifiable T2DM risk factors and the risk of Saudi university students developing T2DM. Second, the modified eight-item AUSDRISK used in this study has not been independently validated in Saudi or Arab young adult populations as a standalone screening instrument; while its individual items are drawn from the validated full AUSDRISK, the psychometric performance of this modified version in the current population is unknown, and this represents a major limitation. Third, the omission of participants’ family history of diabetes from the AUSDRISK computation may have led to a modest underestimation of T2DM risk, particularly in the intermediate risk category. Fourth, BMI was substituted for waist circumference, which may not have fully captured central obesity, a key T2DM risk factor in Arab populations (4). Fifth, physical activity and dietary data were self-reported, making them subject to recall and social desirability bias; the IPAQ short-form in particular is known to overestimate physical activity relative to device-based measures of physical activity (41, 46). Sixth, the predominance of females and undergraduates in the sample limits the generalizability of the findings to the broader Saudi university population. Seventh, sedentary time was estimated by summing self-reported sitting time across six contexts; however, these contexts may co-occur (e.g., mobile phone use while watching television), potentially overestimating total daily sitting time, a recognized limitation of domain-specific sedentary behavior questionnaires. Eighth, the use of an online Google Forms questionnaire distributed via institutional email may have introduced selection bias, as students without reliable internet access or those who did not receive or open the invitation may have been systematically excluded, potentially underrepresenting socioeconomically disadvantaged students and leading to underestimation of T2DM risk prevalence.

## 5 Conclusion

Approximately one in three Saudi university students participating in this multi-regional study had an elevated AUSDRISK-defined T2DM risk. Sex, overweight and obesity, physical inactivity, and lower family income were descriptively associated with elevated AUSDRISK-defined T2DM risk, consistent with their structural role in the scoring algorithm. Among non-AUSDRISK predictors, monthly family income (inversely), smoking status (positively), sedentary behavior, and academic level were all independently associated with elevated AUSDRISK classification in the logistic regression model. The inverse association observed for sedentary behavior should be interpreted cautiously due to potential residual confounding by sex and the structure of the AUSDRISK scoring system. These findings highlight the urgent need to develop and implement sex-specific, socioeconomically targeted T2DM prevention strategies for Saudi university students. Such initiatives should incorporate routine BMI screening, physical activity promotion, and dietary education, particularly for male students from lower-income backgrounds. To confirm the associations outlined in this study between modifiable and non-modifiable T2DM risk factors and inform the development of evidence-based policy to lower the risk of early-onset T2DM among Saudi university students, future researchers should conduct longitudinal studies that include objective metabolic measurements and validated dietary assessments.

## Data Availability

The raw data supporting the conclusions of this article will be made available by the authors, without undue reservation.
