# Supplementary material for: Risk factors for type 2 diabetes mellitus among university students in Saudi Arabia: a multi-regional cross-sectional study
Source: Front Endocrinol (Lausanne). 2026 Jul 28;17:1904706. doi: 10.3389/fendo.2026.1904706 (PMC13457737; doi:10.3389/fendo.2026.1904706)
Supplement: Supplementary file 1 [file DataSheet1.pdf]

STROBE Statement

Checklist of items that should be included in reports of cross-sectional studies

Manuscript title: Risk Factors for Type 2 Diabetes Mellitus Among University Students in Saudi Arabia: A Multi-Regional Cross-Sectional Study

Authors: Abdulrahman I. Alaqil, Fahad Bin Radhyan

Journal: Frontiers in Endocrinology

Item No.	Recommendation	Reported in Manuscript	Page No.
**TITLE AND ABSTRACT**			
**1**	(a) Indicate the study's design with a commonly used term in the title or the abstract (b) Provide in the abstract an informative and balanced summary of what was done and what was found	*(a) 'A Multi-Regional Cross-Sectional Study' stated in the title and abstract (b) Structured abstract provided with Background, Aim, Methods, Results, and Conclusion*	Title; Abstract
**INTRODUCTION**			
**2**	Background/rationale: Explain the scientific background and rationale for the investigation being reported	*Background and rationale provided: Saudi T2DM prevalence (28%), rising burden, gap in multi-regional university studies identified*	Introduction, paras 1–3
**3**	Objectives: State specific objectives, including any prespecified hypotheses	*Aim stated: 'to investigate the association between modifiable and non-modifiable risk factors and T2DM risk among university students across Saudi Arabia'*	Introduction, para 3
**METHODS**			
**4**	Study design: Present key elements of study design early in the paper	*Cross-sectional design stated in Methods section and abstract*	Methods; Abstract
**5**	Setting: Describe the setting, locations, and relevant dates, including periods of recruitment, exposure, follow-up, and data collection	*Setting: Saudi Arabian universities across three regions (Makkah, Eastern Province, Riyadh). Data collection: March–May 2023*	Methods
**6**	Participants: (a) Give the eligibility criteria, and the sources and methods of selection of participants (b) Describe methods of follow-up (N/A for cross-sectional studies)	*(a) University students aged >18 years enrolled at Saudi universities. Systematic random sampling: 3 universities, 3 colleges/university, 2 classes/level/college. Students with prior T2DM diagnosis excluded (n=6). Final n=558. (b) N/A, cross-sectional study*	Methods — Participants
**7**	Variables: Clearly define all outcomes, exposures, predictors, potential confounders, and effect modifiers. Give diagnostic criteria, if applicable	*Outcome: modified AUSDRISK score (0–23) categorized as low (≤5), intermediate (6–11), high (≥12). Predictors: sex, age, BMI, smoking, physical activity (IPAQ-SF), sedentary behavior, dietary intake (FFQ), income, academic level, nationality, marital status. All defined in Methods*	Methods — Measures
**8**	Data sources/measurement: For each variable of interest, give sources of data and details of methods of assessment (measurement)	*Outcome: self-administered modified AUSDRISK questionnaire (Arabic). BMI: self-reported weight/height. PA: IPAQ-SF adapted items. Sedentary: domain-specific sitting time items. Diet: 110-item validated Saudi FFQ. All instruments described*	Methods — Measures
**9**	Bias: Describe any efforts to address potential sources of bias	*Potential biases acknowledged: self-report bias (BMI, PA, diet); social desirability bias; online survey selection bias (email access); response bias (100%*	Methods; Limitations (Eighth)

Item No.	Recommendation	Reported in Manuscript	Page No.
		class-based uptake). Limitations section discusses these	
**10**	Study size: Explain how the study size was arrived at	*Minimum sample size calculated: n=384 (50% expected prevalence, 5% margin of error, 95% CI). Final sample n=564 exceeded this requirement*	Methods — Participants
**11**	Quantitative variables: Explain how quantitative variables were handled in the analyses. If applicable, describe which groupings were chosen and why	*BMI: continuous then categorized (WHO). PA: midpoint calculation → min/week → Low/Moderate/High (WHO). Sedentary: midpoint hours → Low/Moderate/High. Income: ordinal 1–6. AUSDRISK: summed score → Low/Intermediate/High. Rationale for each provided*	Methods — Measures; Statistical Analysis
**12**	Statistical methods: (a) Describe all statistical methods, including those used to control for confounding (b) Describe any methods used to examine subgroups and interactions (c) Explain how missing data were addressed (d) If applicable, describe analytical methods taking account of sampling strategy (e) Describe any sensitivity analyses	*(a) Pearson chi-square, Fisher's exact, independent t-test, one-way ANOVA, binary logistic regression (crude + adjusted OR, 95% CI). Circular regression avoided by excluding AUSDRISK components from regression model (b) Subgroup analysis by sex, BMI, PA — described in Results (c) Complete-case analysis; 6 participants excluded (missing income). <2% missing overall (d) Systematic random sampling described (e) Not performed — noted as future work*	Methods — Statistical Analysis
**RESULTS**			
**13**	Participants: (a) Report numbers of individuals at each stage of study (b) Give reasons for non-participation at each stage (c) Consider use of a flow diagram	*(a) 564 invited and completed survey; 6 excluded (T2DM diagnosis); final n=558 for regression (b) No non-participation — 100% response rate from selected classes (c) Participant flow described in Methods*	Methods; Results
**14**	Descriptive data: (a) Give characteristics of study participants (b) Indicate number of participants with missing data for each variable of interest	*(a) Table 2 presents full sociodemographic and clinical characteristics (b) Missing data minimal (<1.2%); 6 excluded for missing income in regression model*	Results; Table 2
**15**	Outcome data: Report numbers of outcome events or summary measures	*AUSDRISK score: mean 4.68 ± 2.70; range 2–17. Low risk: 67.6%; Intermediate: 29.3%; High: 3.2%. Elevated risk prevalence: 32.4% (n=183). Presented in Tables 3 and 4*	Results; Tables 3–4
**16**	Main results: (a) Give unadjusted estimates and, if applicable, confounder-adjusted estimates and their precision (CI) (b) Report category boundaries when continuous variables were categorized (c) If relevant, consider translating estimates of relative risk into absolute risk for a meaningful time period	*(a) Crude OR and aOR with 95% CI reported for all non-AUSDRISK predictors in Table 5. Income: aOR=0.75 (95% CI 0.61–0.93) (b) Category boundaries reported for BMI, PA, sedentary, and income (c) Not applicable — cross-sectional screening score study*	Results; Table 5
**17**	Other analyses: Report other analyses done — e.g., subgroup analyses, sensitivity analyses	*Subgroup analyses by sex, BMI category, PA level, academic level reported descriptively. No sensitivity analyses performed*	Results; Tables 3–4
**DISCUSSION**			
**18**	Key results: Summaries key results with reference to study objectives	*32.4% elevated AUSDRISK risk. Sex, BMI, PA, and diet associated*	Discussion, para 1

Item No.	Recommendation	Reported in Manuscript	Page No.
		descriptively with elevated risk (structural AUSDRISK components). Income independently associated with elevated classification (aOR=0.75)	
**19**	Limitations: Discuss limitations of the study, taking into account sources of potential bias or imprecision	*Eight limitations discussed: cross-sectional design; unvalidated modified AUSDRISK; family history omission; BMI substitute for waist; self-reported PA and diet; female/undergraduate preponderance; sedentary context overlap; online survey selection bias*	Discussion — Limitations (Eighth)
**20**	Interpretation: Give a cautious overall interpretation of results considering objectives, limitations, multiplicity of analyses, results from similar studies, and other relevant evidence	*Results interpreted in context of AUSDRISK scoring structure. Circularity acknowledged. Comparison with Abd El-Razik Siam et al. (2023), Shiba et al. (2026), Bamogaddam et al. (2023). Income discussed with reference to Agardh et al. (2011) and Yen et al. (2023)*	Discussion, paras 1–7
**21**	Generalizability: Discuss the generalizability (external validity) of the study results	*Generalizability limited by female/undergraduate predominance and restriction to 3 regions. Discussed in Limitations (Sixth)*	Discussion — Limitations
**OTHER INFORMATION**			
**22**	Funding: Give the source of funding and the role of the funders for the present study and, if applicable, for the original study on which the present article is based	*Funded by Deanship of Scientific Research, King Faisal University, Saudi Arabia (Grant No. KFU00000). Funding role: financial support for data collection. No role in study design or interpretation*	Funding section

Note: This STROBE checklist was completed in accordance with the STROBE Statement (Von Elm et al., 2007) for cross-sectional studies. The official STROBE checklist and explanation document are available at www.strobe-statement.org.

Date completed: July 2026

Corresponding author: Abdulrahman I. Alaqil, King Faisal University, Saudi Arabia
